# Characterization of oral microbiome in atopic dermatitis patients treated with dupilumab or upadacitinib

**DOI:** 10.1007/s00784-026-07174-2

**Published:** 2026-10-06

**Authors:** Maciej Wnuk, Justyna Szczęch, Gabriela Żuk, Tomasz Szmatoła, Bernadetta Oklejewicz, Dominik Samotij, Anna Lewińska, Adam Reich

**Affiliations:** 1 Faculty of Biotechnology, Collegium Medicum, University of Rzeszów, Pigonia 1, Rzeszow, 35-310 Poland; 2 Department of Dermatology, Faculty of Medicine, University of Rzeszów, Warzywna 1a, Rzeszów, 35-310 Poland; 3 Department of Animal Molecular Biology, National Research Institute of Animal Production, Krakowska 1, Balice, 32-083 Poland

**Keywords:** Atopic dermatitis, Oral microbiome, Dysbiosis, *Actinomycetota*, EASI

## Abstract

**Objectives:**

Dysbiosis, an imbalanced state of microbial communities in the human body, may contribute to autoimmune diseases such as atopic dermatitis (AD). While the role of affected skin-gut microbiome axis in AD is well established, still little is known about the relationship between oral microflora-based dysbiosis and AD progression and treatment.

**Materials and methods:**

16S rRNA gene amplicon sequencing of the V3–V4 hypervariable region was used to characterize the bacterial profiles of saliva samples from fifteen AD patients treated with dupilumab, an antibody against interleukin-4 receptor, or upadacitinib, an inhibitor of JAK1 kinase, for sixteen weeks. Oral microbiome data were compared with the Eczema Area and Severity Index (EASI).

**Results:**

Analysis revealed that at phylum level, *Actinomycetota* decreased significantly upon treatment (from a median of 11.87% to 6.15%; q = 0.0094), with a reciprocal increase in *Bacteroidota* (from 19.78% to 28.39%; q = 0.0185).

**Conclusions:**

Although all patients showed clinical improvement, the cohort did not permit assessment of a dose–response relationship between EASI score and *Actinomycetota* abundance. Thus, larger, more heterogeneous cohorts and mechanistic data (e.g., metabolomics-based identification of microbiome-derived metabolites) are needed to validate these findings and clarify their relevance as a potential therapeutic marker in AD.

**Clinical relevance:**

The parallel decline in oral *Actinomycetota* abundance and EASI score highlights a potential link between the oral microbiome and treatment response in AD, warranting further investigation. In the absence of non-responders, this association does not yet support *Actinomycetota* as a potential biomarker of treatment efficacy.

**Graphical abstract:**

**Figure:**
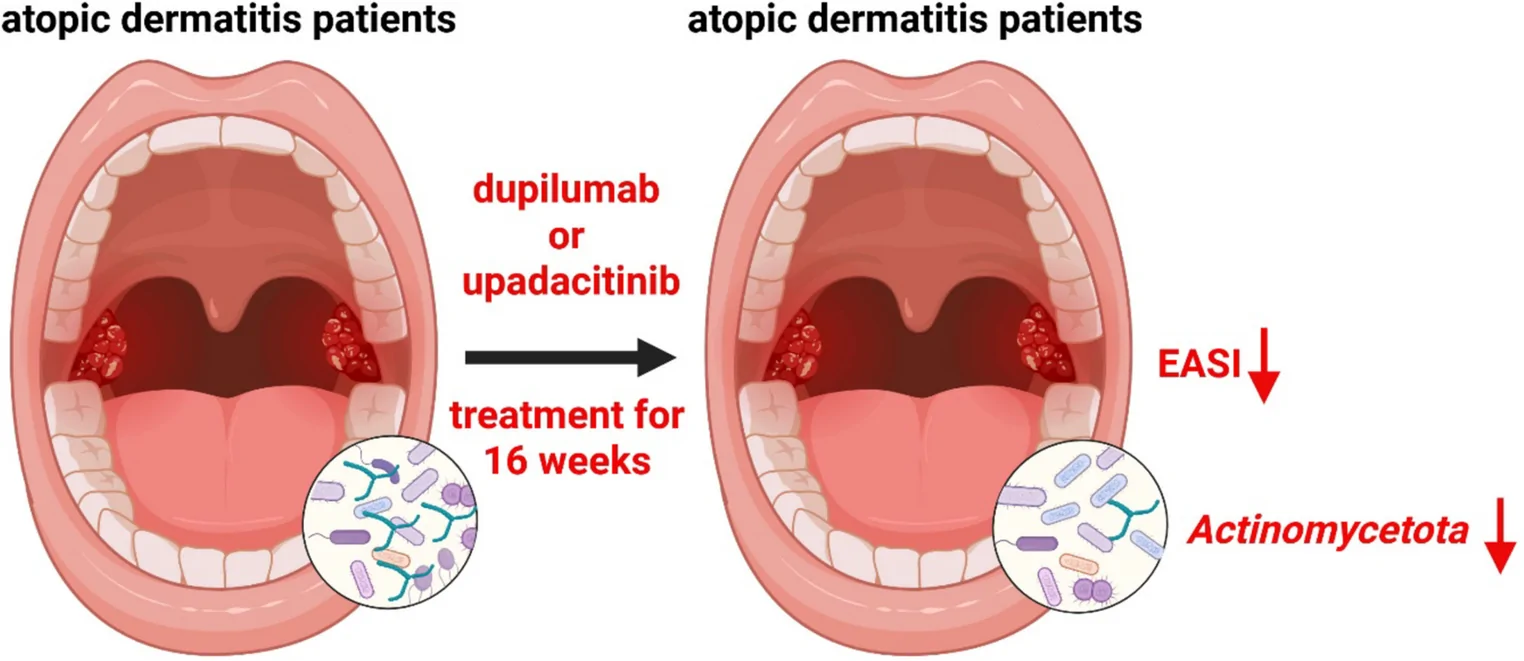

**Supplementary Information:**

The online version contains supplementary material available at https://doi.org/10.1007/s00784-026-07174-2.

## Introduction

Atopic dermatitis (AD), with itching and eczematous lesions, is the most common chronic inflammatory skin disease affecting 15–20% of children and 10% of adults worldwide [1, 2]. The etiology of AD is multifactorial and involves a complex interplay of genetic, immunological, and environmental factors [3, 4]. Genetic predisposition to AD has been well documented. For example, gene mutations compromising skin barrier function, such as mutations in filaggrin gene may contribute to AD development by promoting skin susceptibility to allergens [3, 5]. Indeed, the exposure to various environmental factors, including irritants and pollutants as well as climate changes can also trigger or exacerbate AD [6]. Mutations in selected immunomodulatory genes, such as those encoding for interleukins, e.g., IL-4, IL-13, and interleukin receptors e.g., IL-6R, resulting in the dysregulation of immune system, can also have a significant role in the pathogenesis of AD [7]. Alterations in the skin microflora, e.g., characterized by increased population of *Staphylococcus aureus* and decreased population of *Malassezia* yeasts, and changes in the pools of other pathogenic bacteria or fungi, leading to dysbiosis, are also reported contributors to the etiology of AD [8, 9]. Furthermore, all these factors can act together to potentiate skin inflammation during AD, namely affected skin barrier and microbial communities, when subjected to allergens or pathogens may promote the activation of skin keratinocytes to produce interleukins, e.g., IL-33 and stimulate differentiation of T helper type 2 (Th2) cells secreting IL-4, IL-5, and IL-13 [10–12]. Thus, Th2 inflammation may in turn activate skin keratinocytes and promote secondary proinflammatory response [10–12].

The oral microbiota consists of over 700 opportunistic, commensal, and pathogenic bacterial species belonging to the seven phyla, namely *Actinomycetota* (formerly, *Actinobacteria* or *Actinobacteriota*), *Bacteroidota* (*Bacteroidetes*), *Bacillota* (*Firmicutes*), *Fusobacteriota* (*Fusobacteria*), *Pseudomonadota* (*Proteobacteria*), *Saccharibacteria* (TM7), and *Spirochaetota* (*Spirochaetes*) [13–16]. The most abundant bacterial genera detected in the oral cavity are, among others, Gram-positive cocci (e.g., *Streptococcus*,* Peptostreptococcus*, and *Stomatococcus*), Gram-positive rods (e.g., *Actinomyces*, *Bifidobacterium*, and *Corynebacterium*), Gram-negative cocci (e.g., *Moraxella*, *Neisseria*, and *Veillonella*), and Gram-negative rods (e.g., *Campylobacter*, *Fusobacterium*, and *Prevotella*) [13–16]. The eubiosis of oral microbiota plays a significant role in the maintenance of delicate homeostasis in the oral cavity protecting against colonization of external bacterial pathogens and related diseases [13]. Indeed, common oral disorders such as dental caries, gingivitis, and periodontitis are caused by excessive colonization of bacteria forming biofilms or plaque on teeth [13]. Imbalance in the oral ecosystem due to an increase in the microbial populations or aberrant activity of the immune system can also affect the systemic health [13–17]. The dysbiosis of oral microbiota, infection and chronic inflammation may also stimulate autoimmunological responses by several mechanisms including dysregulation of Toll-like receptors, cytokine-mediated potentiation of autoimmunity, autoantigen complementarity and overproduction [18].

In contrast to a well-established skin-gut microbiome axis in the pathogenesis of AD, data on the role of oral microbiome during AD progression are scarce and contradictory [19–21]. Specifically, current studies report conflicting findings regarding oral microbial diversity: some investigations demonstrate reduced microbial diversity in both children and adult AD patients [19–21], whereas others observe increased diversity in adults with AD compared to non-AD controls [21].

Despite these contrasting findings regarding overall diversity, recent studies point toward consistent alterations in specific bacterial taxa linked to oral health status [21]. For instance, Shahin et al. [21] concluded that AD is associated with oral dysbiosis and poor oral health, as evidenced by increased abundance of taxa related to oral diseases (e.g., *Capnocytophaga ochracea*) alongside decreased abundance of taxa associated with a healthy oral status (e.g., *Haemophilus* and *Gemellaceae*) [21]. Ultimately, more studies are needed to resolve these discrepancies, validate the exact role of oral microbial communities in AD-related pathologies, and explore their potential utility during AD diagnosis and treatment.

In the present study, the oral microbiome of AD patients treated with dupilumab, an antibody against interleukin-4 receptor [22, 23] or upadacitinib, a Janus kinase 1 (JAK1) inhibitor [24, 25] was characterized. We compared the relative abundance of microbial taxa in the oral cavity of AD patients before and after 16 weeks of treatment and examined how these changes are related to clinical improvement. A significant decrease in *Actinomycetota* abundance was revealed upon treatment, accompanied by a reciprocal increase in *Bacteroidota*, suggesting that these phylum-level shifts may reflect treatment-associated changes in the oral microbiome warranting further study.

## Materials and methods

### AD cohort patients, drug treatment, and analyzed clinical biomarkers

**Fig. 1:**
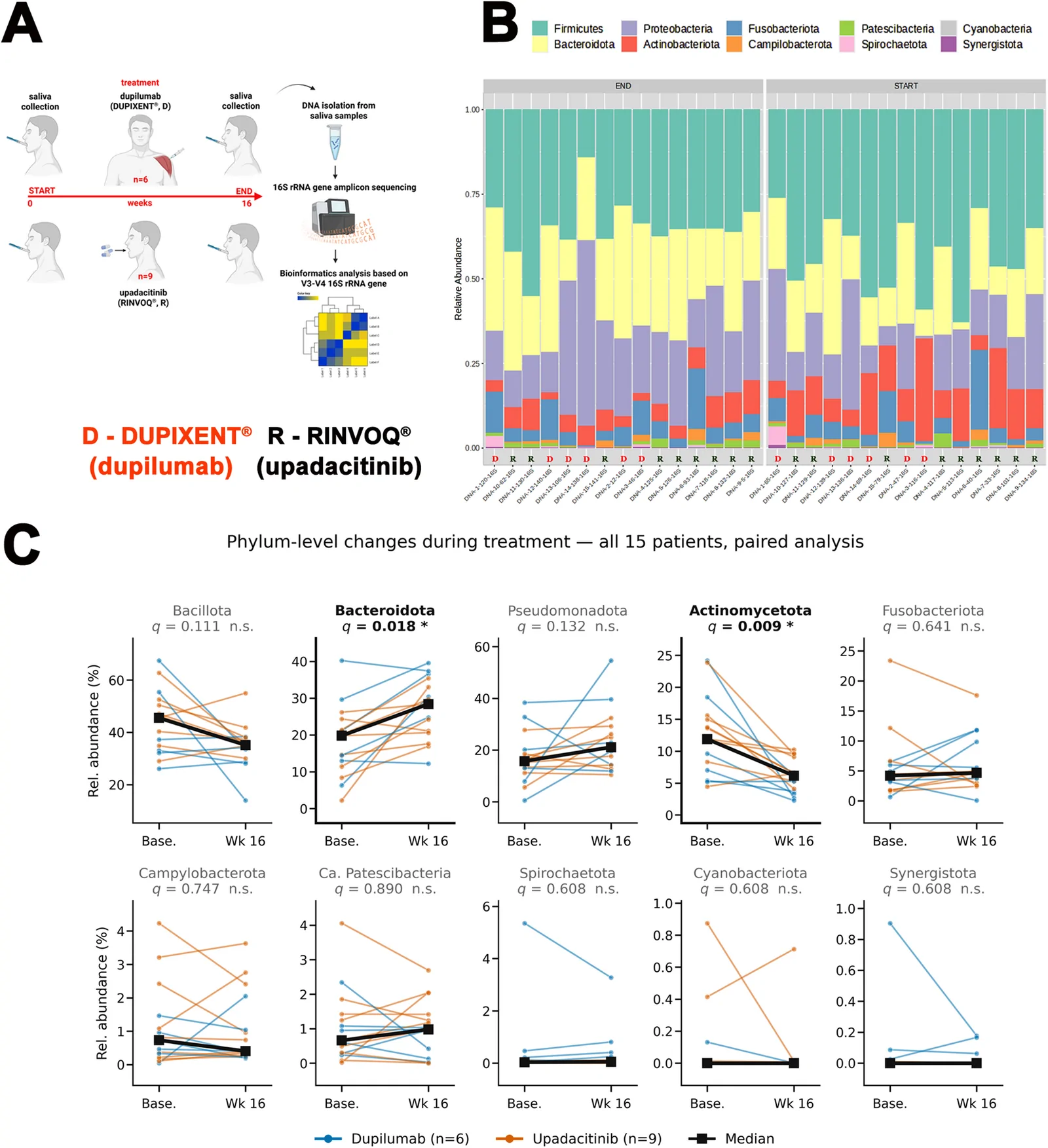
Changes in the abundance of bacterial phyla in the saliva samples from AD patients upon treatment with dupilumab (*n* = 6, subcutaneous) or upadacitinib (*n* = 9, oral administration). (**A**) Study design. Saliva samples were collected from fifteen AD patients before and after treatment for sixteen weeks (START and END points, respectively). 16S rRNA gene amplicon sequencing (V3–V4 region) was used to characterize bacterial profiles in the saliva samples (oral microbiome). (**B**) Relative abundance of ten bacterial phyla in the saliva samples of each individual AD patient coded from 1 to 15. dupilumab treatment cohort (red); upadacitinib treatment cohort (black). (**C**) Relative abundance (%) of the ten most abundant bacterial phyla is shown for each patient at baseline (Base.) and at week 16 of treatment (Wk 16), with connecting lines indicating individual-level change. Line color denotes treatment group: blue, dupilumab (*n* = 6); orange, upadacitinib (*n* = 9). Black squares connected by a bold black line indicate the cohort median (*n* = 15) at each timepoint. For each phylum, the q-value is reported together with its significance status; asterisks (*) denote a statistically significant change relative to baseline (q < 0.05, Wilcoxon signed-rank test with Benjamini–Hochberg correction applied across the ten phyla tested). Relative abundance of *Bacteroidota* increased significantly over treatment (q = 0.018), whereas *Actinomycetota* decreased significantly (q = 0.009); no significant changes were observed for the remaining phyla (*Bacillota*, *Pseudomonadota*, *Fusobacteriota*, *Campylobacterota*, *Ca. Patescibacteria*, *Spirochaetota*, *Cyanobacteriota*, and *Synergistota*; all n.s.)

The study involved fifteen patients who received dupilumab (DUPIXENT^®^, Sanofi Winthrop Industrie, France; six patients) or upadacitinib (RINVOQ^®^, AbbVie Deutschland GmbH & Co, Germany; nine patients) for sixteen weeks (Fig. 1A). The inclusion criteria were as follows: for adults (≥ 18 years of age) – able to consent; for children (> 6 years of age for dupilumab, and  ≥12 years of age for upadacitinib) – legal guardians’ consent; eligible for anti-IL-4R antibody/JAK1 inhibitor treatment due to severity of AD. All drugs were used per label within the approved age range and dosages. Patient characteristics are given in Table 1. Prior to enrolment, all patients underwent a basic physical inspection of the oral cavity. Individuals presenting with clinically overt, major pathological changes—such as active severe caries, advanced periodontitis, or acute gingivitis (corresponding to a Community Periodontal Index * >0*)—were excluded from the study to minimize potential confounding effects of active local infections on systemic inflammatory markers and microbiome profiles. However, it should be noted that a comprehensive, specialist-led dental examination was not performed as part of the study protocol, which represents a methodological limitation regarding the precise evaluation of subclinical oral health status.

Each patient was assessed using the Eczema Area and Severity Index (EASI), i.e., a validated clinical tool for measuring the severity and extent of atopic dermatitis (eczema) in patients, with a final EASI score ranging from 0 to 72. In addition, SCORAD (SCORing of Atopic Dermatitis), Investigator Global Assessment (IGA), and Patient Global Assessment (PtGA: none, mild, moderate, severe, very severe AD) scores were also analyzed [26]. Quality of life was assessed with the Dermatology Life Quality Index (DLQI) [27] and pruritus and cutaneous pain intensity with the Numerical Rating Scale (NRS) [28]. Finally, each patient underwent routine laboratory blood tests, including blood smear, liver function test, C-reactive protein (CRP) levels, total immunoglobulin E (IgE) levels, as per the local guidelines.

Healthy, non-AD volunteers and separate untreated AD control groups were not included in this study, as the primary objective was to perform a longitudinal, paired evaluation of clinical and therapeutic trajectories before and after 16 weeks of targeted systemic treatment. Thus, each patient served as their own internal baseline control, allowing for the precise assessment of treatment-induced changes within and between the two therapy arms.

The study protocol was approved by the Ethics Committee of the University of Rzeszów (057/12/2024).

### Saliva collection, DNA extraction and 16S rRNA gene amplicon sequencing

Saliva samples were collected using sterile nucleic acid-free Eppendorf tubes and stored at -86 °C in an ultra-low temperature freezer until DNA isolation. DNA was isolated using the Wizard Genomic DNA Purification Kit according to the manufacturer’s instructions (A1125, Promega Corporation, Madison, WI, USA). The bacterial composition of saliva samples was characterized by targeted amplicon sequencing of the V3–V4 hypervariable region of the 16S rRNA gene. Shotgun metagenomic sequencing was not performed; consequently, no functional, pathway-level or strain-level inference is reported in this study, and all taxonomic assignments are amplicon-based and subject to the resolution limits of the V3–V4 marker. Targeted 16S rRNA gene amplicon sequencing was applied to characterize the bacterial populations in saliva samples. The V3–V4 hypervariable region of the bacterial 16S rRNA gene was amplified using primers 341 F (5′-CCTACGGGNGGCWGCAG-3′) and 785R (5′-GACTACHVGGGTATCTAATCC-3′), as described by Klindworth et al. (2013) [29], with Illumina overhang adapter sequences appended to each primer (forward overhang: 5′-TCGTCGGCAGCGTCAGATGTGTATAAGAGACAG-3′; reverse overhang: 5′-GTCTCGTGGGCTCGGAGATGTGTATAAGAGACAG-3′), according to the Illumina 16S Metagenomic Sequencing Library Preparation protocol.

Amplicon PCR reactions (25 µl total) contained 2.5 µl of microbial genomic DNA (5 ng/µl in 10 mM Tris, pH 8.5), 5 µl of Amplicon PCR Forward Primer (1 µM), 5 µl of Amplicon PCR Reverse Primer (1 µM), and 12.5 µl of 2x KAPA HiFi HotStart ReadyMix (Roche/KAPA Biosystems). PCR amplification was performed using a thermal cycler under the following conditions: initial denaturation at 95 °C for 3 min, followed by 25 cycles of denaturation at 95 °C for 30 s, annealing at 55 °C for 30 s, and extension at 72 °C for 30 s, with a final extension step at 72 °C for 5 min. Sequencing was performed on a MiSeq instrument using paired-end (PE) technology, 2 × 300nt, with MiSeq Reagent Kit V3 (Illumina).

### Bioinformatic processing

Bioinformatics analysis was performed using QIIME 2 software based on the SILVA 138 reference sequence database. Raw paired-end reads were demultiplexed and imported into QIIME 2 (v2024.10). Primer sequences were removed using cutadapt. Reads were then quality-filtered, denoised, merged and screened for chimeras using DADA2 as implemented in the q2-dada2 plugin. Read quality profiles were inspected to select truncation positions; forward and reverse reads were truncated at 290 and 270 nt respectively, the maximum expected error threshold was set at maxEE = 2 for both reads, reads containing ambiguous bases were discarded, and chimeric sequences were removed using the consensus method. Denoising resolves amplicon sequence variants (ASVs) — exact sequences distinguishable to a single nucleotide — and thereby separates true biological sequences from those introduced by amplification and sequencing error. Denoising performance was monitored using the per-sample read-tracking statistics produced by the q2-dada2 plugin. Across the thirty libraries, a median of 84.1% (range 76.3–89.7%) of input reads was retained after quality filtering and denoising, and a median of 61.8% (range 48.2–71.4%) after merging and chimera removal (Supplementary Material 2 and 4). All samples retained more than 57,000 non-chimeric reads; no samples were excluded at this stage. Taxonomy was assigned to ASVs against the SILVA 138 SSU reference database using the q2-feature-classifier naive Bayes classifier trained on the V3–V4 region extracted with the primer pair used. ASVs assigned to mitochondria or chloroplasts, assigned to *Eukaryota*, or unassigned at domain level were removed prior to downstream analysis. For each PCR reaction performed during library preparation of the V3–V4 16S rRNA amplicons, a no-template negative control and a positive control were included alongside the study samples. Controls were processed in parallel with samples through all subsequent steps of library preparation and sequencing. A batch of libraries was accepted for sequencing only if the negative control yielded no reads or a number of reads below a predefined, negligible threshold; if this threshold was exceeded, library preparation was repeated from the beginning for the entire batch. While negative and positive controls were routinely included at the PCR/library preparation stage to monitor for reagent or cross-sample contamination, no formal bioinformatic decontamination algorithm (e.g., decontam, SourceTracker) was applied to identify or remove specific contaminant taxa from the sequence data. As such, potential low-level contamination that passed the wet-lab quality thresholds cannot be fully excluded, and this should be considered when interpreting low-abundance taxa in the dataset.

### Differential abundance and statistical analysis

For differential abundance analysis, all comparisons were paired, within-subject contrasts between the baseline (START) and week-16 (END) samples of the same patient (fifteen complete pairs). Because subject-level characteristics that are stable over the observation window — including age, sex and baseline disease severity — are identical within each pair, they cannot confound this contrast. Treatment arm, which is not constant across the cohort, was included as a covariate in the models that permit covariate adjustment.

Because differential abundance methods for 16S rRNA gene amplicon data are known to produce divergent results, a consensus approach across multiple methods was adopted as recommended by Nearing et al. (2022) [30]. Four methods were applied: (i) ALDEx2 1.42.0, with 128 Dirichlet Monte-Carlo instances, centered log-ratio transformation and the paired signed-rank test; (ii) ANCOM-BC2 (ANCOMBC 2.12.0), fitted with patient identity as a random intercept and timepoint and treatment arm as fixed effects; (iii) MaAsLin2 1.22.0, with patient identity as a random effect and timepoint, treatment arm, age and sex as fixed effects; and (iv) the Wilcoxon signed-rank test applied to centred log-ratio transformed abundances. Analyses were performed at genus and ASV level on features present in at least 25% of samples at a minimum of 10 reads. Within each method, p-values were corrected across features using the Benjamini–Hochberg procedure, with q < 0.05 considered significant. Taxa significant under at least three of the four methods were defined as the consensus set and form the basis of the conclusions drawn; taxa significant under two methods are reported as suggestive.

**Fig. 2:**
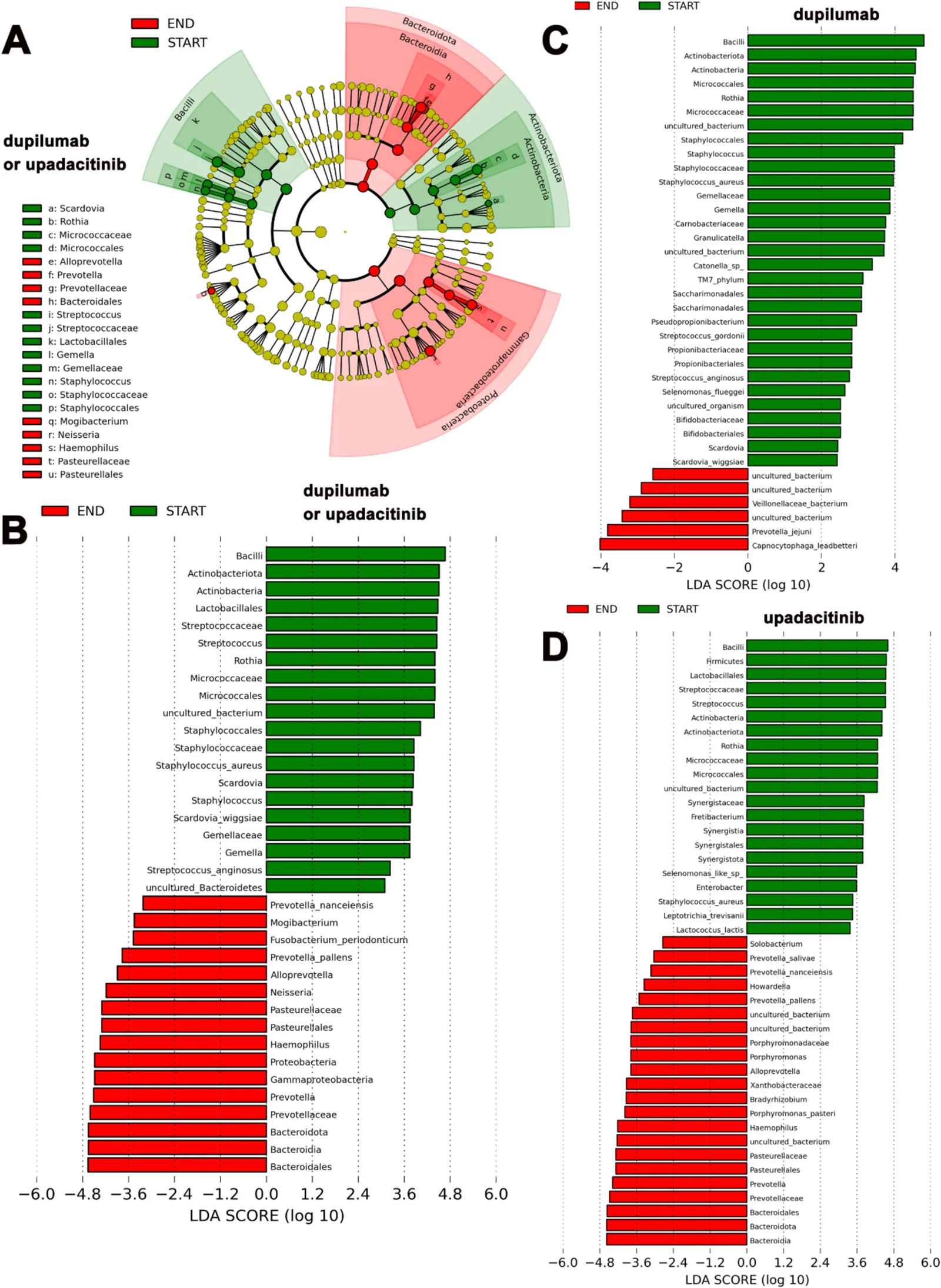
Differential analysis of bacterial profiles in the saliva samples of untreated (START, green) and treated (END, red) AD patients using LEfSe algorithm. (**A**, **B**) Microbiological data upon two treatments (dupilumab and upadacitinib) were combined and analyzed together. (**C**) Analysis upon dupilumab treatment. (**D**) Analysis upon upadacitinib treatment. (**A**) Taxonomic representation (cladogram) of statistically and biologically consistent differences between oral microbiome of untreated (START) and treated (END) AD patients. (**B**, **C**, **D**) LDA score-based plots are shown. LDA score > 2.0 denotes statistically significant differences between bacterial abundance in analyzed microbial communities, here oral microbiome of AD patients before treatment (green) and after treatment (red). The most common phyla, classes, orders, families, genera and/or species are presented

The LEfSe algorithm (Segata et al., 2011) [31] was additionally applied to generate the taxonomic cladogram and LDA effect-size ranking presented in Fig. 2, using the standalone implementation (LEfSe v1.1.2, bioconda) on relative abundance tables exported from QIIME 2 (v2024.10), with the alpha threshold set at 0.05 and an LDA score threshold of 2.0. LEfSe does not model the paired structure of the data, does not allow covariate adjustment, and applies no correction for multiple comparisons across features; its output is therefore presented as a descriptive effect-size summary and is not used as the basis of any inferential claim.

Analyses were performed in R 4.5.3 using ALDEx2 1.42.0, ANCOMBC 2.12.0, MaAsLin2 1.22.0, phyloseq 1.54.0 and rmcorr 0.7.0, and in GraphPad Prism 8 (GraphPad Software, San Diego, CA, USA). The complete per-method output detailing every taxon detected across all algorithms is provided in the Supplementary Material 1 and 4.

## Results and discussion

### Dupilumab and upadacitinib-mediated clinical response in AD patients

Dermatological analysis included fifteen patients with moderate to severe AD treated with either dupilumab (*n* = 6) or upadacitinib (*n* = 9) for sixteen weeks (Table 1). The cohort was characterized by a balanced sex distribution (eight males and seven females) and the patients were predominantly young, with a mean age of 21.8 ± 9.4 years in the dupilumab group (4 adults and 2 pediatric patients aged 10 and 13 years) and 26.2 ± 16.8 years in the upadacitinib group (4 adults and 5 pediatric patients aged 12, 13, 13, 14 and 14 years). Baseline disease severity was high in both groups (Table 1). In patient group treated with dupilumab, the mean EASI was 25.0 ± 4.8 points and the SCORAD was 68.8 ± 10.2 points. In patient group receiving upadacitinib, mean EASI was 23.6 ± 6.2 points and SCORAD was 58.6 ± 6.1 points. Median IGA was 4 (very severe AD) in both groups. Peak pruritus intensity on the numerical rating scale was 8.0 ± 1.4 points in the dupilumab-treated group and 7.7 ± 1.2 points in the upadacitinib-treated group. Health-related quality of life was comparably impaired, with DLQI rated on 15.8 ± 4.1 points for dupilumab-treated group and 16.1 ± 6.5 points for upadacitinib-treated group (an average, very large effect on patient’s life). AD patients were characterized by a strong type 2 inflammation: immunoglobulin E (IgE) levels exceeded 1000 kIU/l in 100% of dupilumab-treated patients and in 88.9% of upadacitinib-treated patients at baseline. Eosinophil levels were modestly elevated in both groups (dupilumab treatment: 0.7 ± 0.3 × 10³/µl; upadacitinib treatment: 0.9 ± 1.0 × 10³/µl). Most patients had low systemic inflammatory activity, with CRP < 0.5 mg/dl in 100% of the dupilumab-treated group and 55.6% of the upadacitinib-treated group. After sixteen weeks of therapy, both treatments led to robust improvement: mean EASI decreased from 25.0 ± 4.8 points to 5.6 ± 3.3 points with dupilumab (mean reduction of 77.6%) and from 23.6 ± 6.2 points to 3.6 ± 3.6 points with upadacitinib (mean reduction of 84.7%). SCORAD declined from 68.8 ± 10.2 points to 27.0 ± 14.7 points with dupilumab treatment and from 58.6 ± 6.1 to 23.1 ± 12.1 points with upadacitinib treatment. Median IGA improved from 4 to 2 in both groups (Table 1). Pruritus improved substantially with both agents; peak pruritus NRS (PP-NRS) fell from 8.0 ± 1.4 points to 4.5 ± 2.8 points among dupilumab treated patients and from 7.7 ± 1.2 points to 3.0 ± 2.2 upon upadacitinib treatment.

**Table 1:** AD patient characteristics before and after treatment with dupilumab or upadacitinib

	Dupilumab, *n* = 6 (2 pediatric patients > 6 to < 18 years)		Upadacitinib, *n* = 9 (5 pediatric patients > 6 to < 18 years)	
Parameter	Baseline	Week 16	Baseline	Week 16
EASI [points]	25.0 ± 4.8	5.6 ± 3.3	23.6 ± 6.2	3.6 ± 3.6
SCORAD [points]	68.8 ± 10.2	27.0 ± 14.7	58.6 ± 6.1	23.1 ± 12.1
IGA [points]	3.8 ± 0.4	2.3 ± 0.5	3.7 ± 0.5	1.7 ± 1.0
PtGA [points]	2.8 ± 1.0	1.0 ± 0.6	2.9 ± 0.8	1.1 ± 0.3
DLQI [points]	15.8 ± 4.1	5.5 ± 2.8	16.1 ± 6.5	3.8 ± 4.6
NRS pruritus [points]	8.0 ± 1.4	4.5 ± 2.8	7.7 ± 1.2	3.0 ± 2.2
NRS pain [points]	5.2 ± 3.9	3.3 ± 2.9	6.8 ± 2.2	2.0 ± 2.3
WBC [×10³/µl]	6.7 ± 1.0	6.5 ± 1.2	7.2 ± 1.9	5.7 ± 1.0
Eosinophils [×10³/µl]	0.7 ± 0.3	0.8 ± 0.4	0.9 ± 1.0	0.3 ± 0.3
Eosinophils (%)	10.9 ± 4.4	12.9 ± 5.9	12.8 ± 11.7	6.0 ± 4.2
IgE total [kIU/l]	> 1000 (100%)	> 1000 (100%)	> 1000 (89%)	> 1000 (78%)
AspAT [U/l]	30.8 ± 7.5	34.5 ± 12.8	29.7 ± 5.3	28.2 ± 4.1
AlAT [U/l]	29.0 ± 11.5	33.7 ± 17.4	25.4 ± 6.8	21.3 ± 4.4
CRP				
Normal [< 0.5 mg/dl]	6 (100%)	6 (100%)	5 (55.6%)	9 (100%)
Elevated [≥ 0.5 mg/dl]	0 (0%)	0 (0%)	4 (44.4%)	0 (0%)

Results are presented as mean±standard deviation. *AlAT* alanine aminotransferase, *AspAT* aspartate aminotransferase, *CRP* C-reactive protein, *DLQI* Dermatology Life Quality Index, *EASI* Eczema Area and Severity Index, *IGA* Investigator Global Assessment, *IgE* immunoglobin E, *NRS* Numerical Rating Scale, *PtGA* Patient Global Assessment, *SCORAD* SCORing of Atopic Dermatitis, *WBC* white blood cells. Individual-level demographic and clinical characteristics of AD patients can be found in Supplementary Material 4.

Quality of life also improved markedly: DLQI declined from 15.8 ± 4.1 to 5.5 ± 2.8 points with dupilumab treatment and from 16.1 ± 6.5 to 3.8 ± 4.6 with upadacitinib treatment. Furthermore, upon upadacitinib treatment, absolute eosinophil counts decreased from 0.9 ± 1.0 to 0.3 ± 0.3 × 10³/µl (Wilcoxon signed-rank test: *p* = 0.02) and eosinophil percentage dropped from 12.8%±11.7% to 6.0%±4.2% (Wilcoxon signed-rank test: *p* < 0.05). In contrast, eosinophil levels upon dupilumab treatment remained stable or slightly increased, rising from 0.7 ± 0.3 to 0.8 ± 0.4 × 10³/µl (Wilcoxon signed-rank test: *p* = 0.75) and from 10.9%±4.4% to 12.9%±5.9% (Wilcoxon signed-rank test:, *p* = 0.75). IgE levels stayed above 1000 kIU/l in all dupilumab-treated patients at week sixteen, whereas the proportion of patients with IgE > 1000 kIU/l in the upadacitinib group decreased from 88.9% to 77.8%, although the difference was not statistically significant. CRP fully normalized in upadacitinib recipients, increasing the proportion with CRP < 0.5 mg/dl from 56% at baseline to 100% at week sixteen (the difference was not significant), while in the dupilumab group the proportion remained unchanged.

Furthermore, several limitations regarding the clinical profile and screening of the study participants should be acknowledged. Although patients underwent a preliminary physical inspection of the oral cavity and those with clinically overt, visible pathological changes (such as active severe caries, periodontitis, or advanced gingivitis) were excluded to minimize confounding systemic inflammatory factors, our study lacked a comprehensive, specialist-led dental examination. The absence of a dental specialist on the research team means that subclinical conditions or minor, non-visible dental caries cannot be entirely ruled out. Given that oral health status and the oral microbiome can potentially interact with systemic inflammation and immune-mediated pathways, future dermatological studies investigating targeted therapies in atopic dermatitis would benefit from incorporating detailed periodontal and dental assessments.

Another limitation of the present study is that the inclusion of both pediatric (aged > 6 years) and adult patients introduces potential confounding variables related to age-dependent physiological variations, such as hormonal profiles and salivary composition. Due to the small sample size (*n* = 15), reliable statistical adjustments or subgroup analyses could not be performed, which should be considered when interpreting the results (Table 1).

### Dupilumab- and upadacitinib-mediated changes in the oral microbiota of AD patients

To evaluate the effect of dupilumab and upadacitinib on the oral microbiome, saliva samples were taken before (START point) and after treatment for sixteen weeks (END point) (Fig. 1A). The oral microbiome was characterized by 16S rRNA gene amplicon sequencing of the V3–V4 region. The detailed study design is shown in Fig. 1A. Amplicon sequencing yielded between 57,000 and 125,000 classified reads per sample, with a classification rate of 100% at the kindom level; at the species level, the proportion of all classified reads ranged between 10% and 25% (see Supplementary Material 2 for more details). The microorganisms belonging to the following ten phyla were distinguished, namely *Firmicutes* (in 2021, renamed to *Bacillota*), *Bacteroidota*, *Proteobacteria* (in 2021, renamed to *Pseudomonadota*), *Actinobacteriota* (in 2021, renamed to *Actinomycetota* but the SILVA label is itself non-standard), *Fusobacteriota*, *Campylobacterota*, *Patescibacteria*, *Spirochaetota*, *Cyanobacteria* (in 2021, renamed to *Cyanobacteriota*), and *Synergistota* (Supplementary Material 2 and 4, and Fig. 1B). Bacterial profiles of saliva samples of each AD patient (before treatment, START, and upon treatment, END; D, dupilumab treatment cohort (in red), upadacitinib treatment cohort (in black)) as relative abundance of all analyzed phyla are shown in Fig. 1B.

When individual patient data were combined across the entire cohort (*n* = 15), the following top five most abundant phyla were identified at baseline: *Bacillota* (median ~ 46%), *Bacteroidota* (median ~ 20%), *Pseudomonadota* (median ~ 16%), *Actinomycetota* (median ~ 12%), and *Fusobacteriota* (median ~ 4%) (Fig. 1C). Upon treatment with dupilumab or upadacitinib, a decreasing trend in *Bacillota* abundance and an increasing trend in *Pseudomonadota* abundance were observed; however, neither change reached statistical significance (q = 0.111 and q = 0.132, respectively) (Fig. 1C). In contrast, *Bacteroidota* abundance increased significantly over the course of treatment (q = 0.018), while *Actinomycetota* abundance decreased significantly (q = 0.009) (Fig. 1C, indicated by asterisks). *Fusobacteriota* abundance remained largely unchanged (q = 0.641) (Fig. 1C). These trends were consistent across both treatment groups, as illustrated by the paired individual-patient trajectories colored by treatment arm (dupilumab, *n* = 6; upadacitinib, *n* = 9) in Fig. 1C.

We have then used LEfSe (*L*inear Discriminant Analysis *Ef*fect *S*iz*e*) approach along with appropriate statistical testing [31] to investigate differences between bacterial groups before and after treatments revealing biological consistency and effect relevance. We have combined data upon treatment with dupilumab or upadacitinib as well as we have analyzed dupilumab or upadacitinib treatments separately (Fig. 2). The use of LEfSe algorithm allows for the identification of significant differences in the bacterial abundance in the saliva samples collected before and after treatment (START vs END) groups of AD patients. The most differentially abundant bacterial groups in the START samples (combined START samples from dupilumab and upadacitinib cohorts) were *Actinobacteriota* phylum *(*in 2021, renamed to *Actinomycetota* but the SILVA label is itself non-standard) and *Bacilli* class from *Firmicutes* phylum (Fig. 2A).

Among *Actinobacteriota* phylum, the most abundant *Micrococcales* order with *Rothia* genus and *Scardovia* genus from *Bifidobacteriales* order can be distinguished. Furthermore, *Bacilli* class was represented by the most abundant *Streptococcus*, *Staphylococcus*, and *Gemella* genera (Fig. 2A). In contrast, upon treatment (dupilumab or upadacitinib treatment), the oral microbiome was characterized by the most differentially abundant group of *Bacteroidota* phylum with *Prevotella* and *Alloprevotella* genera, and *Proteobacteria* phylum with *Haemophilus* and *Neisseria* genera. Along with taxonomic representation, the effect size was also analyzed using linear discriminant analysis (LDA) score (Fig. 2B). *Bacilli* class (*Firmicutes* phylum) and *Actinobacteriota* phylum were the most common in the START samples, whereas *Bacteroidales* order (*Bacteroidota* phylum) was the most common in the END samples. The most differentially abundant bacterial species were *Staphylococcus aureus*, *Scardovia wiggsiae*, and *Streptococcus anginosus* (in the START samples) and *Prevotella pallens*, *Fusobacterium periodonticum*, and *Prevotella nanceiensis* (in the END samples). Relative abundances of three taxa such as *S. aureus*, *S. wiggsiae*, and *S. anginosus* at baseline and after treatment are shown in Supplementary Material 1 and tabulated per sample in Supplementary Material 3. All three decreased significantly (Wilcoxon signed-rank, q < 0.05); for *S. aureus* and *S. wiggsiae*, detection rested on fewer than 100 reads in all but one sample, and these changes are therefore best interpreted as changes in detectability. Although *S. aureus* is widely recognized as a primary colonizer of atopic skin, its detection as a differentially abundant baseline feature in saliva may reflect its presence as a transient member of the oral cavity or a reflection of the participants’ atopic background. To minimize the risk of skin-to-sample contamination, strict standardized sampling protocols were enforced during the study, including the use of sterile collection tools and direct supervision by medical personnel wearing protective gear. Nonetheless, the potential influence of skin-derived transient taxa during saliva collection remains a recognized variable that warrants careful interpretation. When considering dupilumab or upadacitinib treatment alone and effect size, more changes in the abundance of bacterial groups were revealed upon upadacitinib treatment than upon dupilumab treatment (Fig. 2C and D). The most common were the species from *Bacteroidota* phylum, namely *Capnocytophaga leadbetteri* and *Prevotella jejuni* upon dupilumab treatment and *Porphyromonas pasteri*, *Prevotella pallens*, and *Prevotella nanceiensis* upon upadacitinib treatment. When considering the percentage of all classified reads, *Prevotella* genus was one of top three genera identified in almost all END samples (Supplementary Material 2 and 6). *Prevotella* genus was the number one genus in ten END samples with 13% to 29% of all classified reads. Furthermore, *Prevotella histicola* species was detected in six END samples with 1% to 18% of all classified reads (Supplementary Material 2 and 6). It has been previously reported that oral administration of EDP1815, pharmaceutical preparation of non-live *P. histicola* derived from human gut microbiome, may exert protective systemic immunomodulatory action in different preclinical models and clinical trials on inflammation-associated diseases such as multiple sclerosis, psoriasis, and atopic dermatitis as well as during innate and adaptive immune challenge responses [32–35]. For example, treatment with *P. histicola*-based formulation resulted in decreased pools of cytokines related to Th2 or Th17 inflammation, IL-4 and IL-17A, respectively, in mouse models of allergic and/or cutaneous inflammation [33]. Perhaps the presence of *P. histicola* in the oral microbiome may also be associated with improved clinical response of AD patients treated with dupilumab or upadacitinib (this study). However, more studies are needed to document *P. histicola*-mediated anti-inflammatory activity and its effect on dupilumab or upadacitinib-based therapeutic efficacy.

To establish whether the taxa identified were robust to the choice of analytical method, the dataset was re-analyzed using four independent differential abundance methods within a paired, covariate-adjusted framework. At genus level, none of the sixty-seven genera passing the prevalence filter reached a false discovery rate below 0.05 under any of the four methods, either in the pooled analysis or within either treatment arm. The strongest candidates were an increase in *Alloprevotella* (thirteen of fifteen patients; exact paired *p* = 0.0015, q = 0.068) and a decrease in *Rothia* (thirteen of fifteen patients; *p* = 0.0020, q = 0.068), both of which fell just short of significance and are reported here as suggestive only.

At phylum level, the depletion of *Actinomycetota* was confirmed. Across all fifteen patients, *Actinomycetota* relative abundance decreased in thirteen of fifteen individuals, from a median of 11.87% at baseline to 6.15% at week 16 (exact paired Wilcoxon signed-rank test, *p* = 0.0009; q = 0.0094 after Benjamini–Hochberg correction across the eleven prevalent phyla (ten shown)). This change was accompanied by a reciprocal increase in *Bacteroidota*, from a median of 19.78% to 28.39%, which also survived correction (*p* = 0.0034, q = 0.0185). *Bacillota* and *Pseudomonadota* shifted in the directions shown in Fig. 1C but did not survive correction (q = 0.111 and q = 0.132 respectively). Because the two treatment arms comprise only six and nine patients, the arm-specific comparisons are underpowered and are reported as exploratory; the depletion of *Actinomycetota* was directionally consistent in both arms (eight of nine patients receiving upadacitinib, five of six receiving dupilumab) (Supplementary Material 4 and 5).

Taken together, these analyses indicate that the phylum-level shift is robust, whereas the genus- and species-level differences identified by LEfSe should be regarded as hypothesis-generating and require confirmation in a larger cohort.

Phylum-level relative abundances were compared between the baseline and week-16 samples using the *Wilcoxon* signed-rank test, which accounts for the paired structure of the data. Eleven prevalent phyla were tested; p-values were corrected for multiple comparisons using the Benjamini–Hochberg false discovery rate procedure, and q < 0.05 was considered significant.

As mentioned above depletion of *Actinomycetota* was evident at phylum level, where relative abundance fell from a median of 11.9% to 6.1% and decreased in 13 of 15 patients (Wilcoxon signed-rank *p* < 0.001, q = 0.005; Supplementary Material 1), however among individual *Actinomycetaceae* members, both *Actinomyces* (genus, excluding *Schaalia*) and *Schaalia* showed a directional but non-significant decrease (*p* = 0.229 and *p* = 0.507 respectively; Supplementary Material 1 and 3), indicating that the phylum-level shift reflects the aggregate behaviour of several low-abundance taxa rather than the depletion of any single genus.

The relationship between disease severity and *Actinomycetota* abundance was assessed in two ways. First, the common within-patient association between EASI score and *Actinomycetota* relative abundance was estimated by repeated-measures correlation (rmcorr) [36], which accounts for the non-independence of the two observations contributed by each patient. Second, the within-patient change in EASI between baseline and week 16 was correlated with the corresponding change in *Actinomycetota* relative abundance across the fifteen patients using Spearman’s rank correlation coefficient; this analysis is based on fifteen independent observations and is not affected by the parallel temporal decline in both variables.

**Fig. 3:**
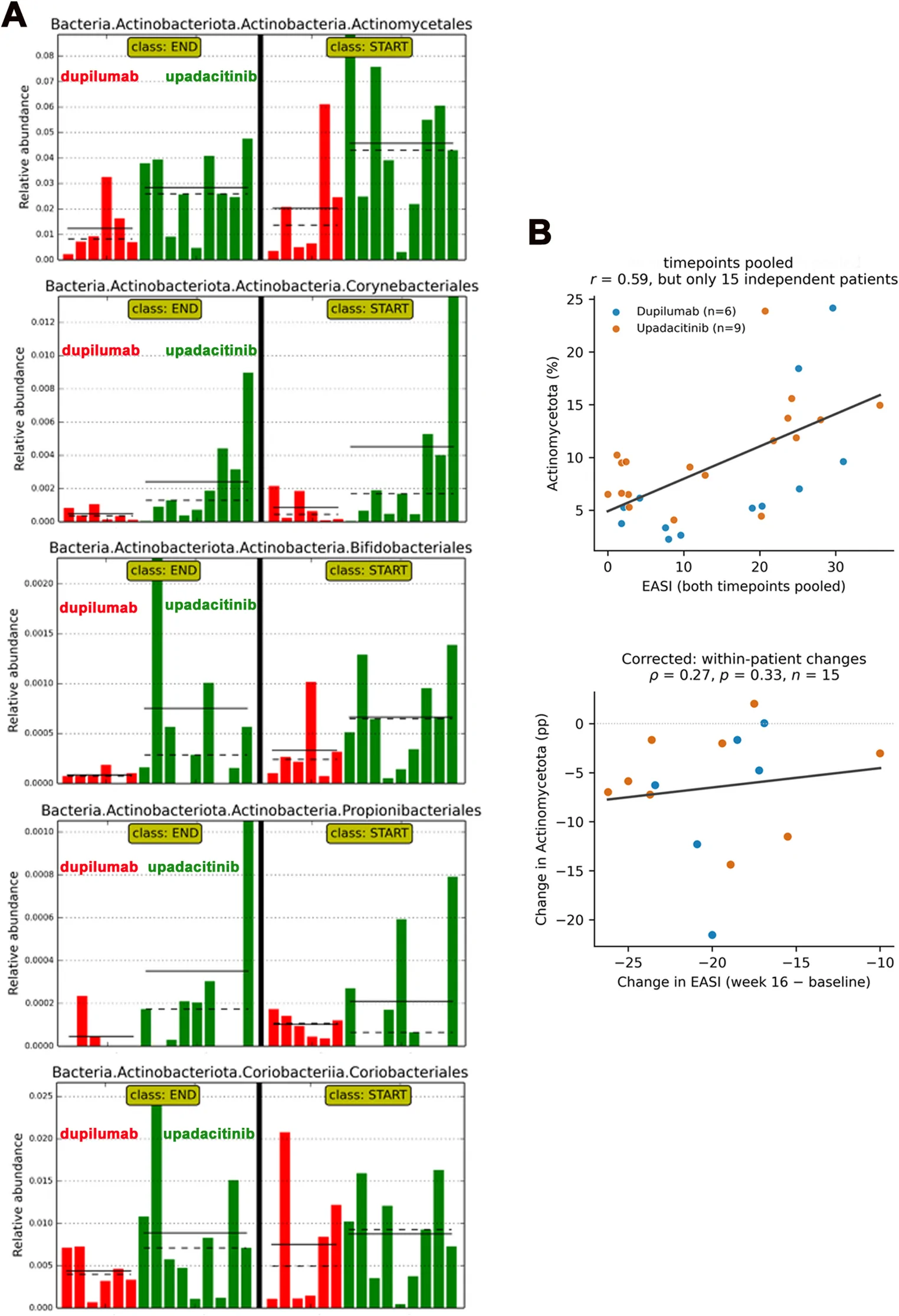
Actinobacteriota-phylum taxa dynamics and their relationship to clinical improvement in atopic dermatitis patients treated with dupilumab or upadacitinib. (**A**) Relative abundance of five orders within the phylum *Actinobacteriota* (*Actinomycetota*) — *Actinomycetales*, *Corynebacteriales*, *Bifidobacteriales*, *Propionibacteriales*, and *Coriobacteriales* — shown separately for each patient at baseline (“START”) and at week 16 (“END”). Bars represent individual patients treated with dupilumab (red) or upadacitinib (green); the solid horizontal line indicates the group mean and the dashed line the group median. Across all five orders, relative abundance is generally higher at baseline than at week 16, consistent with a treatment-associated depletion of *Actinobacteriota*-phylum taxa during successful AD therapy. (**B**) Relationship between clinical improvement (EASI) and *Actinomycetota* relative abundance, before and after statistical correction. Top panel - timepoints pooled. *Actinomycetota* relative abundance plotted against EASI score, with baseline and week-16 measurements pooled together (*n* = 15 patients × 2 timepoints = 30 points; dupilumab, blue, *n* = 6; upadacitinib, orange, *n* = 9). This pooled analysis yields *r* = 0.59, but because each patient contributes two non-independent observations, the true number of independent data points is 15, not 30. Moreover, since both EASI and *Actinomycetota* abundance decline in parallel between baseline and week 16, pooling the two timepoints produces a positive correlation even if no genuine within-patient relationship between disease severity and microbiota exists — the correlation largely reflects the shared temporal trend rather than a true dose–response relationship. Bottom panel - corrected analysis (within-patient change scores). Change in *Actinomycetota* relative abundance (percentage points, week 16 minus baseline) plotted against change in EASI (week 16 minus baseline), using one independent data point per patient (*n* = 15). This analysis directly tests whether patients with greater clinical improvement also showed a greater reduction in *Actinomycetota* abundance. The corrected association is weak and not statistically significant (Spearman’s ρ = 0.27, *p* = 0.33; Pearson *r* = 0.13, *p* = 0.64), indicating that the apparent correlation in the pooled analysis was driven by the parallel decline of both variables over time rather than by a genuine coupling between clinical response and microbiota change

As dupilumab or upadacitinib treatment resulted in a statistically significant decrease in bacterial populations classified to the *Actinobacteriota* phylum in the saliva samples (Fig. 1C), we decided then to analyze more comprehensively this bacterial taxonomic group and their members in each individual AD patient. Changes in the populations of bacteria from *Actinomycetales* and *Corynebacteriales* (*Mycobacteriales*) orders from *Actinobacteriota* phylum may contribute to observed decrease (Fig. 3A and Supplementary Material 1). Indeed, two species from *Actinomyces* genus (i.e., *Actinomyces graevenitzii* and *Actinomyces pacaensis*) and *Schaalia odontolytica* (formerly *Actinomyces odontolyticus*) were identified as the most common *Actinobacteriota* (*Actinomycetota*) members in the START saliva samples of individual AD patients in terms of the percentage of all classified reads (Supplementary Material 2 and 6). A positive correlation between AD manifestation as judged by EASI score and the presence of *Actinobacteriota* (*Actinomycetota*) (%) was initially observed using Pearson correlation coefficient-based analysis on pooled START and END timepoints (*r* = 0.677, *p* = 0.015 for the dupilumab treatment cohort, and *r* = 0.602, *p* = 0.0082 for the upadacitinib treatment cohort). However, this analysis pools two timepoints per patient and is therefore confounded by the shared temporal trend, since both EASI score and *Actinobacteriota* (*Actinomycetota*) abundance decreased over the course of treatment in nearly all patients. When the analysis was corrected to test the relationship between the magnitude of clinical improvement and the magnitude of change in *Actinobacteriota* (*Actinomycetota*) abundance within each patient (change in EASI score vs. change in *Actinomycetota* abundance, week 16 minus baseline, *n* = 15), no significant association was found (Spearman’s rho = 0.271, *p* = 0.328; Pearson’s *r* = 0.134, *p* = 0.635) (Fig. 3B and Supplementary Material 4 and 5). This indicates that although *Actinobacteriota* (*Actinomycetota*) abundance declines during treatment, the extent of this decline does not track the extent of clinical improvement, and the originally reported correlation reflects the parallel decline of both variables over time rather than a dose–response relationship (Fig. 3B and Supplementary Material 4 and 5). Although mechanistic conclusions cannot be drawn due to the lack of clinical oral health indices, these correlations may point towards an association between oral *Actinobacteriota* (*Actinomycetota*) shifts and AD severity. Furthermore, alterations in these microbial populations might hold potential as exploratory biomarkers for monitoring therapeutic responses, though longitudinal studies controlling for oral health status are required to validate these hypotheses.

The present study has several limitations. First, the cohort is small (fifteen patients; six receiving dupilumab and nine receiving upadacitinib), and the arm-specific analyses in particular are underpowered and are reported as exploratory and hypothesis-generating. Second, although the paired design means that patient characteristics stable over sixteen weeks cannot confound the baseline-versus-week-16 contrast, body mass index was not recorded, and the study includes no untreated control arm; we therefore cannot formally separate a treatment effect from temporal drift in the oral microbiome, or from changes in diet, oral hygiene or concomitant medication over the sixteen-week interval. Third, differential abundance methods for 16S rRNA gene data are known to yield divergent results (Nearing et al., 2022) [30]; we have mitigated this by adopting a consensus framework across four methods, but the consensus set nonetheless requires replication in an independent cohort. Fourth, V3–V4 amplicon sequencing does not reliably resolve oral bacteria to species level, and species-level assignments should be regarded as provisional. Fifth, oral microbiome-related mechanisms remain to be investigated; the oral microbiome-associated metabolome should be characterized to identify metabolites potentially responsible for AD progression or attenuation, with particular emphasis on metabolites produced by *Actinomycetota*, as the oral pool of this phylum was the most affected upon treatment in the present study.

There are three reports on the characterization of oral microbiota in AD patients [19–21], however with no emphasis on the role of the *Actinomyces* and *Schaalia* genera from the *Actinomycetota* phylum. To the best of our knowledge, changes in the bacterial profiles in the oral cavity of treated AD patients have not yet been investigated. Thus, the present study is the first analysis of dupilumab- and upadacitinib-mediated effects of oral microbiome and related clinical response in AD patients. *Actinomyces* and related microorganisms such as *Schaalia*, commensal oral microbiota members responsible for cervicofacial actinomycosis, may also contribute to the development of selected dental infections such as caries, root canal infection, periapical infection, and periodontitis [37]. For example, *A. viscosus* and its lipoproteins can stimulate the Toll-like receptor 2 (TLR2)-dependent production of IL-8 and tumor necrosis factor alpha from human gingival epithelial cells and macrophages promoting periodontal inflammation [38].

As atopic dermatitis was reported to be associated with impaired oral health and oral dysbiosis [21], one can suggest that *Actinomyces* and related metabolite-mediated oral infections may also promote the manifestation of inflammation-associated atopic dermatitis. The effects of selected metabolites produced by commensal bacteria found in atopic dermatitis lesions have been already documented [39]. For example, short chain fatty acids (SCFAs) such as acetate, propionate, and butyrate may modulate immune function and maintain epidermal homeostasis by the regulation of the nuclear factor kappa-light-chain-enhancer of activated B cells (NF-κB) activity, cytokine and nitric oxide production, the attenuation of IgE-mediated allergic responses, the inhibition of histone deacetylation in keratinocytes to suppress inflammation, and the reduction of *S. aureus* colonization in epidermis [39]. It would also be worthwhile to explore the effects of metabolites produced by oral microbiota on AD manifestation and therapeutic outcomes.

As already mentioned the present study has several limitations. First, a larger cohort of AD patients is required to characterize the effects of dupilumab and upadacitinib on the oral microbiome more comprehensively and with greater statistical power. Second, because all patients in this cohort showed clinical improvement, the study design did not allow us to test for a dose–response relationship between the magnitude of microbial change and clinical outcome; cohorts including partial or non-responders will be needed to establish whether these microbial shifts track treatment efficacy rather than merely accompanying it. Third, the underlying oral microbiome-related mechanism(s) remain to be investigated — in particular, the oral microbiome-associated metabolome should be characterized to identify metabolites potentially responsible for AD progression and/or attenuation, including their immunomodulatory activity, both pro- and anti-inflammatory. Given that *Actinomycetota* was the phylum most consistently affected by dupilumab and upadacitinib treatment in this study, we propose that future research place particular emphasis on characterizing the metabolites produced by this phylum, alongside the reciprocal increase observed in *Bacteroidota*.

## Supplementary Information

Below is the link to the electronic supplementary material.

Supplementary Material 1 (ZIP 2.61 MB)

## Data Availability

Data supporting the findings of this study are available in Supplementary Material 1 to 6.
