# Supplementary material for: Characterization of oral microbiome in atopic dermatitis patients treated with dupilumab or upadacitinib
Source: Clin Oral Investig. 2026 Oct 6;30(11):511. doi: 10.1007/s00784-026-07174-2 (PMC13642382; doi:10.1007/s00784-026-07174-2)
Supplement: Supplementary file 1 [file 784_2026_7174_MOESM1_ESM.zip › Supplementary Material/Supplementary_legend.docx]

**Supplementary_Materials 1.docx**

- **Table S1** – Wilcoxon signed-rank test results (paired, baseline vs. week 16) for six key taxa, with median (IQR), detection rates, direction of change, P, BH-adjusted q, and rank-biserial effect size r.
- **Figure S1** – Volcano-style scatter of −log₁₀(q) across four differential-abundance methods (Wilcoxon-CLR, ALDEx2, MaAsLin2, ANCOM-BC2) for 67 genera, plus a Spearman rank-concordance heatmap between methods.
- **Figure S2** – Paired patient trajectories of *Actinomycetota* and *Bacteroidota* relative abundance, baseline vs. week 16.
- **Figure S3** – Paired trajectories of *S. aureus*, *S. wiggsiae*, *S. anginosus* relative abundance (log scale), with detection status.
- **Figure S4** – Paired trajectories of *Actinomyces* (excl. *Schaalia*) and *Schaalia* relative abundance.
- **Figure S5** – Phylum-level *Actinomycetota* (*Actinobacteriota*) relative abundance shown for context alongside its genus-level constituents.

**Supplementary_Materials 2 (Supplementary_Material_taxonomy_over_samples.xlsx)**

Raw taxonomic count tables for all 30 saliva 16S rRNA (V3–V4) libraries (15 patients × 2 timepoints), one sheet per taxonomic rank: Kingdom, Phylum, Class, Order, Family, Genus, Species. Each sheet is a feature table with full lineage strings as row labels and per-sample read counts as columns (samples named e.g. "DNA-1-65-16S").

**Supplementary_Materials 3 (Supplementary_relative_abundance_key_taxa.xlsx)**

Relative-abundance dataset for the six taxa. Sheets:

- **README** – purpose statement.
- **Read_counts** – raw per-sample read counts for the six key taxa plus total classified reads.
- **Relative_abundance** – same taxa expressed as % of total classified reads per sample.
- **Paired_by_patient** – relative abundance at W0/W16 arranged side-by-side per patient.
- **Summary_statistics** – per-taxon medians/IQR, patients increased/decreased/unchanged, detection change, log2FC, Wilcoxon P, BH-adjusted q, rank-biserial r, exact detection P.
- **By_treatment_arm** – same summary statistics split by dupilumab vs. upadacitinib.
- **Arm_comparison** – Mann–Whitney U test comparing within-patient log2 fold-changes between the two arms.
- **Sensitivity_analysis** – P-values recomputed at increasing minimum read-count thresholds (≥0, ≥5, ≥10, ≥20 reads) to test robustness.

**Supplementary_Materials 4 ( Supplementary_Tables_S1_S6.xlsx)**

Numbered table workbook matching the manuscript's Table S1–S6 scheme. Sheets:

- **README** – index of all six tables plus Fig. S2.
- **S2_nomenclature** – same SILVA→current-name mapping as in the .docx.
- **S3a_genus_pooled / S3b_genus_dupilumab / S3c_genus_upadacitinib** – genus-level differential abundance (four methods), pooled and per arm.
- **S4_phylum_tests** – phylum-level paired tests, pooled and per arm.
- **S6_EASI_Actinomycetota** – per-patient EASI and *Actinomycetota* data (same as in the DA reanalysis file).
- **S7_Individual-level demographic and clinical characteristics of AD patients before (Week 0) and after (Week 16) treatment with dupilumab or upadacitinib.**

**Supplementary_Materials 5 (Supplementary_DA reanalysis.xlsx)**

Consensus differential-abundance re-analysis. Sheets:

- **README** – purpose and dataset description (30 libraries, 15 patients, dupilumab n=6 / upadacitinib n=9).
- **S4_phylum** – paired phylum-level tests (pooled), with n pairs, direction, medians, P, q.
- **S3_genus_pooled / S3_genus_dupilumab / S3_genus_upadacitinib** – genus-level differential abundance from four methods (ALDEx2, ANCOM-BC2, Wilcoxon-CLR, MaAsLin2) with effect sizes, P/q values, consistency flag, and a summary "call" (significant/ns), for the pooled cohort and each treatment arm separately.
- **S6_EASI_vs_*Actinomycetota*** – per-patient EASI scores (baseline/end) and *Actinomycetota* relative abundance (%), with deltas.

**Supplementary_Materials 6 (Supplementary_Table_Group_Mean_Relative_Abundance.xlsx)**

- **Sample_Group_Key**
- **Data_Kingdom / Data_Phylum / Data_Class / Data_Order / Data_Family / Data_Genus / Data_Species**
- **MeanRelAb_Kingdom / MeanRelAb_Phylum / MeanRelAb_Class / MeanRelAb_Order / MeanRelAb_Family / MeanRelAb_Genus / MeanRelAb_Species**.
