# Supplementary material for: Characterization of oral microbiome in atopic dermatitis patients treated with dupilumab or upadacitinib
Source: Clin Oral Investig. 2026 Oct 6;30(11):511. doi: 10.1007/s00784-026-07174-2 (PMC13642382; doi:10.1007/s00784-026-07174-2)
Supplement: Supplementary file 1 [file 784_2026_7174_MOESM1_ESM.zip › Supplementary Material/Supplementary_Material 1.docx]

### **Supplementary relative-abundance dataset — oral microbiome, atopic dermatitis**

**Table S1** Two-sided Wilcoxon signed-rank test on paired relative abundances (zero differences dropped); q = Benjamini–Hochberg FDR across the six taxa; r = matched-pairs rank-biserial correlation (negative indicates a decrease after treatment); ↓ / ↑ = number of patients in whom the taxon decreased / increased

| **Taxon** | **Baseline W0**  **median (IQR), %** | **Post-treatment W16**  **median (IQR), %** | **Detected** | **↓ / ↑** | **P** | **q (BH)** | **r** |
| --- | --- | --- | --- | --- | --- | --- | --- |
| *Staphylococcus aureus* | 0.0060 (0.0000–0.0120) | 0.0000 (0.0000–0.0000) | 9/15 → 2/15 | 7 / 2 | **0.021** | **0.042** | -0.87 |
| *Scardovia wiggsiae* | 0.0106 (0.0063–0.0198) | 0.0073 (0.0000–0.0107) | 13/15 → 9/15 | 12 / 1 | **0.016** | **0.042** | -0.76 |
| *Streptococcus anginosus* | 0.0831 (0.0294–0.1569) | 0.0426 (0.0200–0.0731) | 13/15 → 14/15 | 10 / 5 | **0.030** | **0.045** | -0.63 |
| *Actinomyces (genus, excl. Schaalia)* | 2.47 (0.69–4.33) | 1.84 (0.62–3.22) | 15/15 → 15/15 | 9 / 6 | 0.229 | 0.275 | -0.37 |
| *Schaalia (S. cardiffensis + S. odontolytica)* | 0.1310 (0.0000–0.7549) | 0.1193 (0.0609–0.1843) | 10/15 → 13/15 | 7 / 6 | 0.507 | 0.507 | -0.21 |
| Actinobacteriota (phylum) | 11.87 (7.67–15.27) | 6.15 (3.91–7.85) | 15/15 → 15/15 | 13 / 2 | **<0.001** | **0.005** | -0.90 |

Values in bold are significant at q < 0.05. Full per-sample data, per-arm results and the read-depth sensitivity analysis are in the accompanying workbook.

Read counts were converted to relative abundance as the number of reads assigned to a taxon divided by the total number of classified 16S rRNA gene reads for that sample, expressed as a percentage. Baseline (W0) and post-treatment (W16) values were compared within patients using two-sided Wilcoxon signed-rank tests, with zero differences excluded; P values were adjusted across taxa by the Benjamini–Hochberg procedure. Effect size is reported as the matched-pairs rank-biserial correlation. Change in detection was tested with an exact binomial test on the numbers of patients gaining and losing detection. Analyses were performed in Python 3 (SciPy 1.17, statsmodels). Treatment arms were compared by Mann–Whitney U test on within-patient log₂ fold changes; no taxon differed between arms (all P > 0.20), and arms were therefore pooled.

**Supplementary Figure S1**. No genus-level taxon reaches statistical significance after correction for multiple testing, and rank concordance among the four differential abundance methods. (a) Scatter plot of −log₁₀(q) values for all 67 genera passing the prevalence filter, shown separately for each of the four methods applied to the pooled dataset: paired Wilcoxon signed-rank test on centred log-ratio (CLR) transformed abundances, ALDEx2, MaAsLin2, and ANCOM-BC2. The red dashed line indicates the significance threshold (q = 0.05). No genus reached q < 0.05 under any method. The two strongest candidates, *Alloprevotella* (increased in 13 of 15 patients) and *Rothia* (decreased in 13 of 15 patients), are highlighted in orange; both achieved the lowest q-values under the paired Wilcoxon–CLR analysis (q = 0.068, ranked first and second respectively) but fell short of the significance threshold and are therefore reported as suggestive rather than as confirmed findings. (b) Heatmap of Spearman correlation coefficients computed on genus-level q-value ranks, quantifying pairwise rank concordance between methods. Values range from 0 (no concordance) to 1 (perfect concordance). Agreement was highest between the paired Wilcoxon–CLR test and MaAsLin2 (ρ = 0.79) and between Wilcoxon–CLR and ALDEx2 (ρ = 0.76), whereas ANCOM-BC2 showed consistently lower concordance with the other three methods (ρ = 0.31–0.37), reflecting its distinct underlying statistical assumptions.

**Supplementary Figure S2.** Phylum-level shifts in *Actinomycetota* and *Bacteroidota* relative abundance from baseline to week 16. Paired individual-patient trajectories of relative abundance (%) for (left) *Actinomycetota* and (right) *Bacteroidota*, shown at baseline and week 16 for all fifteen patients, coloured by treatment arm (blue: dupilumab, n = 6; orange: upadacitinib, n = 9). Black squares connected by a bold line indicate the median at each timepoint. Actinomycetota abundance decreased in 13 of 15 patients, from a median of 11.87% at baseline to 6.15% at week 16 (exact paired Wilcoxon signed-rank test, q = 0.0094 after Benjamini–Hochberg correction across eleven prevalent phyla), while *Bacteroidota* abundance increased reciprocally in 12 of 15 patients, from a median of 19.78% to 28.39% (q = 0.0185). Both changes remained significant after correction for multiple testing; the depletion of *Actinomycetota* was directionally consistent in both treatment arms (5/6 dupilumab, 8/9 upadacitinib patients), although arm-specific comparisons are underpowered given the small sample sizes.

**Supplementary Figure S3**. Relative abundance (%, log scale) of *Staphylococcus aureus* (A), *Scardovia wiggsiae* (B) and *Streptococcus anginosus* (C) at baseline (W0) and post-treatment (W16). Lines connect paired samples from the same patient; colour denotes treatment arm (dupilumab, n = 6; upadacitinib, n = 9). Open symbols on the “0” row below the axis break indicate samples with no reads assigned to the taxon; the number of patients in which the taxon was detected is given beneath each group. P values are from two-sided Wilcoxon signed-rank tests on paired relative abundances.

**Supplementary Figure S4**. Relative abundance (%, log scale) of *Actinomyces* (genus level, excluding *Schaalia*) (A) and *Schaalia* (*S. cardiffensis* + *S. odontolytica*) (B) at baseline and post-treatment. Symbols, colours and statistics as in Figure S1. Neither taxon changes significantly at the paired patient level.

**Supplementary Figure S5**. Relative abundance (%, log scale) of the phylum *Actinomycetota* (*Actinobacteriota*) at baseline and post-treatment, shown for context. The depletion is significant and consistent across patients (13/15 decreased), in contrast to its individual *Actinomycetaceae* constituents.
